# Decoding corporate communication strategies: Analysing mandatory published information under Pillar 3 across turbulent periods with unsupervised machine learning

**DOI:** 10.1371/journal.pone.0328841

**Published:** 2025-07-31

**Authors:** Anna Pilková, Michal Munk, Lívia Kelebercová

**Affiliations:** 1 Comenius University in Bratislava, Bratislava, Slovakia; 2 Science and Research Centre, University of Pardubice, Pardubice, Czech Republic; 3 Department of Computer Science, Constantine the Philosopher University in Nitra, Nitra, Slovakia; Kitami Institute of Technology, JAPAN

## Abstract

This study explores the communication patterns of Slovak banks with stakeholders through mandatory disclosures mandated by Basel III’s Pillar 3 framework and annual reports in 2007−2022. Our primary objective is to identify key topics communicated by banks and analysing the sentiment of this communication during turbulent periods (i.e., alternating periods of stability and crisis) in 2007−2022. Textual data was collected from Pillar 3 disclosures, annual reports, and additional regulatory reports. A hybrid model was developed to extract the most important keywords from each collected document chapter. This hybrid model (model combining multiple approaches) combines elements of statistical approaches to keyword extraction, (keyword frequency dictionary), linguistic approaches (pair-of-speech tagging in order to select noun-phrases), and machine-learning based approaches (BERT) to extract meaningful keywords. Subsequently, a sentiment analysis was performed on the extracted keywords using a Loughran-McDonald lexicon (list of words labelled with sentiment) specially designed for financial texts. Based on the adjusted univariate results, we can reject the global null hypothesis of independence of the sentiment category of keywords from time for negative sentiment at *p* = 0.0000 for positive sentiment at *p* = 0.0005, and for neutral sentiment at *p* = 0.0000 significant level. The multilevel comparison revealed that negative sentiment was most frequent during the global financial crisis and the COVID-19 pandemic, likely impacting stakeholder confidence and trust. Conversely, positive sentiment dominated during periods of financial stability, potentially enhancing stakeholder satisfaction and investment decisions. This research points out that the sentiment of the selected commercial bank documents changes depending on the years. A commercial bank can use this knowledge and include sentiment information as predictors when modelling financial distress. For bank management of selected commercial bank the examined documents are an important communication tool, the wording of which can have a significant impact on stakeholder behaviour towards the bank, their styling is very important.

## Introduction

The Basel Framework, introduced by the Basel Committee on Banking Supervision – the primary global standard setter for the prudential regulation of banks, is a comprehensive regulatory framework. Its Basel III framework was designed to address the limitations of its predecessors Basel I and Basel II in calculating capital requirements, supervisory review and disclosures, and aims to respond to the lessons learnt from the Global financial crisis in 2008-2009 (GFC) which was triggered by the collapse of the housing market in the United States and the subsequent failure of major financial institutions, leading to a global economic downturn. The GFC revealed that previous standards needed strengthening, particularly because banks had excessive leverage and insufficient liquidity buffers, combined with governance and risk management issues. A new version of framework serves a comprehensive package of reviews of capital and liquidity standards, introduced in stages between 2013 and 2019, and includes an enhanced framework for disclosure requirements, referred to as Pillar 3.

Pillar 3 is dedicated to market discipline, recognizing the pivotal role that informed investors and stakeholders play in maintaining financial stability. It emphasizes the need for financial institutions to disclose key information about their risk profile, capital adequacy, and risk management practices to enable market participants to make informed decisions. The pillar imposes specific disclosure requirements to ensure that financial institutions provide comprehensive information regarding their risk exposures, risk management processes, and capital adequacy. This includes the disclosure on the composition of capital and exposures to the main risks (credit risk, market risk, counterparty credit risk, operational risk, securitization positions and others), and disclosures related to other parts of the regulatory framework, such as regulatory liquidity ratios, leverage ratio, asset encumbrance, remuneration or interest rate risk in the banking book. Market transparency enables market participants to assess the institution’s risk profile and supports them in making informed investment decisions. The disclosure requirements encompass both qualitative and quantitative aspects, including information on risk governance, risk concentration, and the outcomes of stress testing. This multifaceted approach enables a holistic understanding of a bank’s risk management framework.

Effective communication and transparency are essential for building trust and ensuring market stability, especially during times of financial uncertainty. Understanding stakeholder sentiment through these disclosures is crucial, as it reflects the market’s perception of a bank’s stability and risk management effectiveness. Sentiment analysis of these disclosures can provide insights into how stakeholders might react to the information provided, influencing their investment decisions and overall market behaviour.

With the evolution and strengthening of the Basel III framework, the Pillar 3 requirements were gradually adjusted between January 2015 and December 2018, mirroring the new areas of regulation added to the Basel Framework. The adjustments were necessary to address the evolving risks and challenges in the financial sector, including the need for better risk management practices and increased transparency. The recent version of the Pillar 3 standard and templates has been in use since January 1, 2022. Pillar 3 is also a part of the latest version of regulatory framework (Basel IV). It emphasizes the importance of timely and consistent reporting to facilitate accurate and up-to-date assessments of a bank’s financial health. This ensures that market participants have access to relevant information for making informed decisions. Timely and consistent reporting is especially important these days, given that financial crises are increasingly common in the market, and in times of financial uncertainty, market discipline is a mechanism for the functioning of the market and the prevention of financial difficulties for its main players. Financial crises, such as the GFC, or COVID-19 pandemic, have highlighted the importance of robust risk management and transparent communication to maintain market confidence and stability. Disclosure information is considered one of the most effective tools for improving market discipline. In general, the implementation of Pillar 3 is a set of requirements related to appropriate disclosure of information that allows market participants to assess key information leading to the ability to make more informed investments.

In the case of Slovak banks, the qualitative information part of the Pillar 3 framework is included in the reports called “Information on the bank’s activities,” which in its recent form has been available quarterly since 2020 and includes textual information relevant to this study.

In addition to the prudential requirements disclosures under Pillar 3, banks also publish their annual reports. The annual reports have the regulatory basis is the accounting rules, and aim to provide the relevant stakeholders (e.g. shareholders, investors and depositors) with information about bank’s strategy, market conditions, and future outlook. Historically, annual reports primarily focused on financial performance, providing a snapshot of a company’s economic health. However, with the introduction of frameworks like Basel III and increasing demands for transparency, these reports have become more comprehensive. These reports include both financial statements and narrative sections that discuss the Annual reports have evolved significantly over time, reflecting changes in regulatory requirements, technological advancements, and stakeholder expectations. Annual reports and information from the prudential disclosure under Pillar 3 provide a comprehensive set of information on financial, operational and risks related aspects in banks.

Banks, as issuers of shares and bonds, fall also under capital market related regulations, under which they disclose various reports as well. The most relevant are the Yearly financial reports, Semi-annual financial reports, and covered bonds prospectus(for banks issuing covered bonds). Examining the sentiment of bank communications during turbulent periods can uncover patterns and trends that are critical for investors, analysts, and regulators. Over the years, banks have faced various turbulent periods. A period of stability can alternate from year to year with a period of crisis. These crises can be triggered by various factors, including economic downturns, geopolitical events, and market failures, which can lead to significant changes in the banking sector. Understanding how banks communicate with stakeholders at different times provides valuable information about their strategies, challenges, and necessary adaptations. In order to find out how banks communicate information, a text analysis of financial reports through which banks communicate with end users can be used. By analysing these reports, we can obtain information about the sentiment or tone of these reports, which in principle means that we can examine whether the information contained in bank reports has a positive, negative or neutral character. The goal of our study is to identify key topics that selected commercial Slovak bank communicated through documents related to mandatory disclosure within the Pillar 3 from 2007 to 2022. The period 2007-2022 was chosen because the commercial bank had documents publicly available on its website from 2007 to 2022. In each year, we will identify the most important topics from individual documents using a summarization technique called keyword extraction. We will analyse the extracted keywords from the point of view of sentiment and then look at whether banks communicated their key information in a positive, negative or neutral way in individual years, taking into account the ongoing social, economic and geopolitical events. The examined periods are the periods before the GFC (2007-2008), the period of GFC (2009-2010), after the GFC (2011-2016), after the revision of pillar 3 (2017-2019) and during the challenges brought by the COVID-19 pandemic (2020-2021), or the energy crisis and the war in Ukraine (2022). The COVID-19 pandemic brought unprecedented challenges to the global economy, including supply chain disruptions, widespread lockdowns, and a shift in consumer behaviour, all of which had significant impacts on the banking sector. Similarly, the energetic crisis and the war in Ukraine have led to increased volatility in energy prices and geopolitical risks, affecting banks’ operations and risk management strategies. Does the sentiment of the key topics communicated by the Slovak commercial bank in the documents depend on the year in which these documents were published? The importance of this study lies in its potential to identify patterns and trends in corporate communication during moments of economic uncertainty. Sentiment analysis can reveal to stakeholders how various aspects of a bank are perceived, such as its financial performance, reputation, customer satisfaction, innovation or risk management. If sentiment changes among years, it can be used as an indicator of the bank’s future performance. For example, if investor sentiment towards a bank is positive, it may indicate that they expect growth and success. Conversely, negative sentiment can be a warning sign of trouble. Due to our long-standing cooperation with Slovak banks, we selected documents from a selected commercial bank for our research, which are publicly available on its website. The rest of the paper is structured as follows. The Related works on sentiment in mandatory disclosure documents section provides a comprehensive overview of research on sentiment analysis in annual reports, with a particular focus on studies related to banking. The following section named Approaches to keyword extraction describes the fundamentals behind the most-known keyword extraction methods. The section Approaches for sentiment analysis in financial domain discuss advantages and disadvantages of machine learning based approaches and lexicon based approaches. Materials and methods describes the process of data collection, creation of keyword extraction model, post-processing of extracted keywords and the way sentiment was analysed from those keywords. Results section contains the results of a statistical analysis, the aim of which was to verify whether the sentiment of the keywords presented in the bank’s disclosures changed depending on the investigated periods. The results are further discussed in the sections entitled Discussion.

## Related works

The presented study follows on from our previous experiments conducted at Slovak banks, which did not directly deal with sentiment analysis, but analysed whether the turbulent periods that Slovak banks are going through have an impact on stakeholders’ interest in mandatory disclosures under Pillar 3. The following sections of the paper are devoted to a brief description of these studies.

The overarching objective of our previous research was to investigate fluctuations in stakeholder interest. Specifically, we focused on Pillar 3 information during periods of economic turbulence in Slovakia. One of our studies [1] focused on analysing the effectiveness of Pillar 3 disclosures within commercial banks during market turbulence. This investigation leveraged web server log files from commercial banks operating in Slovakia, encompassing data from 2009 to 2015, with a particular emphasis on quarterly analysis. The methodology involved rigorous data preprocessing techniques, including data cleaning, integration, transformation, session identification, path completion, and data reduction, to ensure the log files were adequately prepared for analysis. The findings revealed a heightened interest in disclosures immediately following the 2009 GFC, which subsequently waned overtime.

Our subsequent study [2] delved deeper into examining stakeholder interest in Pillar 3 disclosures, particularly during significant market turbulence. This research aimed to evaluate the extent to which current banking regulations support stakeholders’ interest in the information mandated by regulators for disclosure. Additionally, it sought to understand stakeholder behaviour and assess the effectiveness of Pillar 3 disclosures as a market discipline tool. The study utilized web server log files from two major commercial banks in Slovakia, with data spanning from 2009 to 2015 for the first bank and from 2016 to 2018 for the second bank. These periods covered both the GFC and post-GFC eras. The log files were meticulously preprocessed to create variables such as time (quarter, year) and web categories (e.g., Pillar 3 disclosure requirements, annual reports, group information). The analysis yielded compelling conclusions: post-2009, stakeholder interest in disclosed information declined, and no significant temporal trends or seasonality in stakeholder behaviour regarding Pillar 3 information were observed after 2012.

Collectively, these studies, which examined data from Slovak banks from 2009 to 2018, unequivocally demonstrate that global economic changes exerted a profound impact on the Slovak banking sector. The insights gleaned from these investigations underscore the dynamic interplay between economic conditions and stakeholder engagement with regulatory disclosures. Subsequent to these studies, we decided to examine not only the log files of Slovak commercial banks but also the documents themselves published under Pillar 3 and annual reports. Building on the related studies listed below, the sentiment expressed in these papers could help us model financial distress in future research.

A seminal study [3] aimed to explore the potential of sentiment analysis in detecting risks within the banking system by analysing textual data from bank annual reports. The primary goal is to determine if sentiment analysis can serve as a tool for banking supervision, particularly in predicting changes in quantitative risk indicators like the Tier 1 Capital Ratio (T1). The study used over 500 CEO letters and outlook sections extracted from annual reports of 27 Eurozone banks between 2001 and 2013. Tier 1 Capital Ratio (T1), which measures a bank’s ability to absorb losses was used as quantitative indicator. After obtaining the text data basic preprocessing steps as word-tokenization, removing punctuation, numbers, and stop words removing were used. The authors used lexicon-based approach (finance-specific word list to tag words) and also machine learning based approach (assigning classed UP or DOWN based on the evolution of the Tier 1 Capital Ratio and using Naïve Bayes, Support Vector Machines classifiers) to assign sentiment class. In case of lexicon-based approach, sentiment scores reflected major economic events and showed strong correlations with the Tier 1 Capital Ratio evolution. Regression models using sentiment scores could predict Tier 1 Capital Ratio changes, but only when data were aggregated by year. Speaking of the machine learning-based approach, classification data accurately predicted the direction of Tier 1 Capital Ratio changes for 12 out of 13 years. Following limitation have been found. The model cannot account for external shocks like regulatory changes or monetary policy actions. Predictions for individual banks were less accurate compared to aggregated data. The study suggests that sentiment analysis can provide valuable insights for macroprudential analyses and improve existing risk prediction frameworks, but it should be used in conjunction with other estimation methods.

A notable study [4] aimed to investigate the relationship between public sentiment and banking crises. The researchers aimed to understand how shifts in public sentiment, as reflected in various media and social platforms, could serve as early indicators or influencers of financial instability within the banking sector. The study utilized a diverse dataset comprising sentiment analysis from social media platforms, news articles, and financial reports. The data spanned several years, including periods of financial stability and instability, to capture a comprehensive range of sentiment fluctuations. The researchers also incorporated historical banking data, such as stock prices, trading volumes, and economic indicators, to correlate sentiment trends with actual banking events. The collected text data required the same preprocessing steps as in the case of [3]. Predefined lists of words with associated sentiment scores (positive, negative, or neutral) were used to evaluate the sentiment of each token. Sentiment scores for individual texts were aggregated over time to create a sentiment time series. This allowed researchers to observe trends and fluctuations in public sentiment over the study period. The sentiment scores were then aggregated over time to create a sentiment time series. This was compared with a time series of banking sector performance indicators. Regression models and machine learning algorithms were used to identify correlations and causal relationships between sentiment trends and banking crises. The models were validated using historical data and tested for predictive accuracy. The study found a significant correlation between negative public sentiment and subsequent banking crises. Periods of heightened negative sentiment were often followed by declines in banking sector performance, including drops in stock prices and increased volatility. The results suggested that sentiment analysis could provide valuable insights for predicting financial instability. In general the findings in accordance with previously mentioned study [3]. They both highlighted the potential of sentiment analysis in predicting financial indicators and crises. The findings have practical applications for risk management and regulatory policy in the banking industry.

An interesting study by Dong et al. [5] was examining the annual reports covering the years 2004 to 2016 through the textual analysis. They gathered 2053 bank reports, and 372 documents published by the Bank for International Settlements. Their preprocessing steps were same as in aforementioned studies. Subsequently they made counted the frequency of appearance of words in the reports focusing on those words that appeared in at least 10% of the reports. Loughran and McDonald used a dictionary to analyse sentiment. The authors concluded that the trend in textual dynamics is closely associated with regulatory requirements. While disclosed information can predictably be linked to future earnings and liquidity risk of the bank, the standardization of disclosure in Pillar 3 has also led to less individualistic bank characteristics, as texts have become increasingly homogeneous. While their study offered valuable insights, it did not explore the relevance of communicant topics or the evolution of sentiment in turbulent economic periods. Our study addresses this gap by focusing on the sentiment dynamics across multiple economic periods, providing a more comprehensive understanding of bank communication strategies.

An interesting study [6] investigates whether the narrative sections of annual reports, specifically the Management Discussion and Analysis (MD&A) sections, can predict a company’s financial performance. Same preprocessing steps were needed. The study analysed 20 companies from the Fortune 1000 list, divided into financially distressed and stable groups based on Altman’s Z-score. For sentiment analysis, lexicon-based approach was used to determine sentiment of each word in narratives using LIWC2015 software. The interesting finding is that financially distressed companies used a different tone in their annual reports. The sentiment in annual reports changed as companies approached bankruptcy. As a limitation, we can say that the study analysed a limited number of companies, which may affect the reliability and generalizability of the findings.

Building up to this study the authors tried to bring more focus to sentiment fluctuations among years 1995-2018 [7]. Each annual report was analysed individually for the mentioned sentiment categories. The analysis focused on the frequency and proportion of words related to each sentiment category. There were noticeable fluctuations in positive sentiment over the years, with sharp increases in 2008 and 2009, possibly to present a more optimistic outlook during financial difficulties. The study found that companies may use more positive language during financially challenging times to reassure stakeholders. As the limitation we can point out that the study focused solely on IBM, which may limit the generalizability of the findings to other companies or industries.

Another study by Kubaščíková et al., [8] examines sections containing non-financial information from annual reports of Slovak, Turkish and Ukrainian Companies. The data source came from 400 annual reports from companies in the three countries in 2008-2018. The main objective of the study was to analyse the sentiment expressed in annual reports from companies in Slovakia, Turkey, and Ukraine, and its correlation with the countries’ GDP growth. Annual reports were again analysed using LIWC2015 to determine the percentage of words reflecting different emotions. The analysis focused on how the tone and structure of language in annual reports changed over time, alongside economic growth. In case of the Ukraine negative emotions prevailed in Ukrainian annual reports when GDP was expected to decrease. The sentiment reflected the impact of geopolitical conflicts, such as the Ukrainian-Russian conflict, on business operations. For Turkey the correlation between emotions in annual reports and GDP growth was less obvious but still present. Positive emotions generally prevailed, but the growth or decrease of these emotions did not always align with GDP trends. For Slovakia, positive emotions in Slovak annual reports correlated with economic growth. The sentiment reflected the economic stability and growth.

Studies focusing on Slovakia have provided compelling insights into how Slovak banks navigated economic turbulence, as reflected in sentiment and stakeholder behaviour. Research on Pillar 3 disclosures during the GFC revealed heightened stakeholder interest immediately following the 2009 crisis, indicating a response to economic uncertainty [1]. However, this interest declined over time, suggesting adaptation and stabilization within the banking sector. Another study [2] further supported these findings, noting that while stakeholder interest in disclosures peaked during the crisis, it subsequently waned, with no significant trends post-2012, reflecting a return to normalcy. Additionally, Kubaščíková et al. [8] found that positive emotions in Slovak annual reports correlated with economic growth, indicating that sentiment in bank communications mirrored the country’s economic stability and recovery.

From a methodological point of view, the above studies had several common features. The first common feature is that they performed sentiment analysis on documents that are published within Pillar 3 or annual reports in order to determine whether there is an association between the sentiment expressed in these documents and turbulent times or the financial stability of the company. We share this common objective with them. All of these studies were demanding in terms of data preparation. Data preparation required word tokenization, punctuation removal, punctuation removal, digit removal, stop word removal. In the case of inflected languages, it would also have to include lemmatization. Sentiment was determined for individual words and their thematic/keyness was not taken into account. Our approach differs from the aforementioned studies. We created a model that performs the aforementioned data preprocessing steps in the background. The model extracts keywords from the given texts according to the desired range of n-grams and according to the desired diversity. This way, we can ensure that we extract from the documents phrases that represented the central theme in the given chapters of annual reports or documents related to mandatory disclosure and at the same time are not too similar. We then evaluate the sentiment of only these keywords. We then use lexicons, such as some of the studies mentioned above, to analyse sentiment. We consider the practical contribution of our work to be the creation of a simpler methodology for analysing the sentiment of complex documents that are demanding in terms of data preparation. We consider the results of the statistical analysis, which we describe in the Results section, to be beneficial to the application domain. The selected commercial bank can use the results to analyse the historical contexts of its documents to adjust communication with stakeholders in the future. Given the statistical significance of differences in sentiment depending on whether the bank is going through a crisis or not, we consider it appropriate to consider adding information about sentiment as a predictor for modelling the bank’s financial performance.

## Approaches to keyword extraction

Keyword extraction plays a significant role in identifying the most relevant terms and phrases from documents. The idea of the concept of keywords or key phrases consists in capturing the content of the document or its selected part in order to capture the concept of the examined document. Keyword extraction approaches might be divided into statistical approaches, linguistic approaches, machine learning approaches, or hybrid approaches which consist of combinations of the mentioned approaches [9].

### Statistical approaches

Statistical approaches involve of quantitative methods for identifying keywords. Statistical methods for keyword extraction don’t need prior examples to learn. They rely on basic text statistics, like how often words appear, to find important phrases. These methods are quick and work well across different languages. However, the identified phrases might not always be meaningful or relevant [9].

**TF-IDF** (Term Frequency-Inverse Document Frequency) is a widely recognized method belonging to statistical methods for identifying important words within a document. It assigns weights to terms based on their frequency in a document relative to their frequency in the entire corpus. This technique is particularly effective for languages like English, where word forms are relatively stable and consistent. Term Frequency (*TF*): This measures how frequently a term appears in a document. The more a term appears, the higher its TF value. Inverse Document Frequency (*IDF*): This measures how important a term is by comparing its frequency across all documents in the corpus. Terms that are common across many documents (like “the” or “and”) get lower *IDF* values, while rare terms get higher values. The TF-IDF score is then calculated by multiplying the *TF* and *IDF* values. This ensures that terms which are frequent in a document but rare in the corpus receive higher weights, indicating their importance. Inflective languages, such as Slovak, Czech, or Polish, have rich morphological systems where words change forms based on grammatical context. In inflective languages, a single word can appear in many different forms. For example, the Slovak word “*dom*” (house) can appear as “*domu*,” “*domom*,” “*domov*,” etc., depending on the grammatical context. TF-IDF treats each of these forms as distinct terms, diluting the importance of the root word. To accurately measure term importance in inflective languages, lemmatization is often necessary to reduce different word forms to their base form. In case of selecting more complex phrases (bigrams, trigrams, etc.) it can lead to selecting phrases without meaning (context). TF-IDF is also used in more complex statistical keyword extraction methods, such as **KP-Miner** [10], which implements two criteria in addition to TF-IDF. The first criterion is the lowest allowed frequency of vision. This condition ensures that a word becomes a candidate if its occurrence in the document exceeds a specified frequency threshold. The second criterion is the boundary position of the word within the document. Another algorithm using TF-IDF is **YAKE** [11,12], which combines it with local context analysis. YAKE is an unsupervised method that considers text statistical properties like word casing, position, frequency, and co-occurrence to extract keywords.

Another possible way is to use the frequencies of word collocations, which is the principle of the **RAKE** (Rapid Automatic Keyword Extraction) statistical method. RAKE uses word co-occurrence and frequency within a sliding window to identify phrases that are likely to be keywords. It focuses on contiguous sequences of words and assigns weights based on word frequency and degree of co-occurrence. This approach can be more effective than TF-IDF for Slovak, as it considers the context and co-occurrence of words, which helps mitigate the issues arising from the language’s rich morphology. However, RAKE still may not be good enough for Slovak because it does not inherently handle the variability of word forms caused by inflections. Without proper lemmatization and stop words filtering, RAKE might still treat different forms of the same word as distinct entities, leading to dispersed importance scores and potentially inaccurate keyword extraction [13,14].

### Linguistic approaches

Linguistic approaches rely on the grammatical structure and meaning of words to identify key terms. They analyse words, how they are used in sentences, and their overall meaning. These methods use rules related to word types (like nouns, verbs, adjectives), word combinations (like phrases of two or more words), and grammatical chunks (like noun phrases). For example, they can specifically look for phrases that follow the pattern of “adjective + noun” (e.g., significant improvement). A major advantage of these methods is that they tend to extract phrases that make sense and are relevant to the topic. However, a drawback is that they often require specific rules for each language, making it difficult to apply them across different languages [9].

A popular method that can be classified as linguistic method (sometimes also classified as graph based method) as it uses part of speech tagging is the **TextRank** method, which is based on the PageRank algorithm [15,16]. Candidate words are filtered based on their parts of speech and connections between them are made through co-occurrence relationships. If two words fall within a moving window of parameterized size, they are considered connected and form a directed graph where nodes represent candidate words. PageRank is then used to calculate word scores. In other words TextRank represents words or phrases as nodes in a graph and edges as co-occurrence relationships. Keywords are identified based on the importance of nodes in the graph. While it has advantages over TF-IDF in some aspects, it also faces challenges when applied to inflective languages. Similar to TF-IDF, TextRank struggles with the variability of word forms in inflective languages. Different inflections of the same word are treated as distinct entities, which can disperse the importance score among these variants. To mitigate the issues stemming from word form variability, TextRank requires preprocessing steps like lemmatization. Another problem could be the graph constructed by TextRank that might become highly complex and dense due to the numerous word forms, making the algorithm computationally intensive and less efficient.

### Machine learning approaches

Machine learning approaches are relatively new trend that has only been developing for the last few years. Unsupervised methods, by their nature, are often more adaptable to different domains. Since they do not rely on a specific training set, they can generalize well to new text datasets from various domains, unlike supervised methods, which may overfit to the specific labelled dataset they were trained on. Many real-world applications involve unstructured data that is difficult to annotate manually. Unsupervised keyword extraction is well-suited to handle this unstructured data without needing prior human intervention or supervision, making it a practical choice for scalable text analysis. One of the key techniques used in machine learning approaches for keyword extraction is embeddings. Embeddings are high-dimensional vector representations of words or phrases that capture semantic meaning and contextual relationships. Models like BERT (Bidirectional Encoder Representations from Transformers) generate these embeddings by training on large corpora of text, allowing them to understand and represent the language. By leveraging embeddings, machine learning approaches can identify and extract semantically meaningful keywords that are more relevant and contextually appropriate. Approaches using machine learning can effectively extract meaningful keywords, but they are language-dependent and computationally demanding [9].

### Hybrid approaches

Hybrid keyword extraction approaches combine the strengths of aforementioned approaches to enhance the relevance of extracted keywords. These methods usually integrate measures, such as term frequency (from statistical approaches), part-of-speech tagging (from linguistic approaches), embeddings (from machine learning approaches), etc. to effectively extract keywords from domains [9].

Supervised machine learning approaches for keyword extraction typically leverage labelled training datasets, such as collections of social media posts annotated with relevant keywords. These methods often employ traditional machine learning algorithms like Support Vector Machines and Naïve Bayes. While supervised methods can be effective, unsupervised learning approaches have gained prominence due to their ability to identify keywords independent of specific domains and training data. Unsupervised methods often fall under the umbrella of hybrid approaches. They typically involve a multi-stage process consisting of generating candidate phrases using statistical or linguistic techniques, constructing vector representations of these candidate phrases and the target document, calculating the similarity between each candidate phrase and the document to identify the most representative keywords. A hybrid approach **EmbedRank** [17] works by determining candidate phrases based on a sequence of parts of speech and then calculating the cosine similarity between the vector of the document and the vector of the candidate phrase.

The **KeyBERT** technique [18,19] works in a similar way. However, it uses BERT embeddings for representations and uses statistical methods based on frequencies of occurrence to generate candidate phrases. BERT is an effective technique, but it is mainly intended for short documents. Maximal Marginal Relevance is used to select top n keywords. It is a ranking algorithm that selects items (e.g., keywords or sentences) by balancing relevance to the main query/document and diversity among the selected items. It works iteratively, scoring candidates based on their relevance to the input while penalizing those similar to already-selected items. This approach ensures that the selected keywords or sentences are both highly relevant and non-redundant, making it particularly useful in summarization and keyword extraction tasks.

Another technique for short documents using embeddings is **SIFRank** [20]. This technique combines SIF embeddings and the ELMo language model. The authors also presented an optimized model for long documents called **SIFRank+** [20]. Approaches based on machine learning can extract semantically more meaningful phrases compared to statistical approaches, but the disadvantage is language and domain dependence and computational complexity. On the other hand, machine learning-based approaches usually require less data preprocessing.

A challenge in the field of keyword extraction methods are inflectional languages, such as Slovak language, where words can differ in endings. A possible solution would be lemmatization before the extraction process, but in the case of multi-word phrases the meaning would be lost. One of the contributions of the presented study is the design of the architecture of a hybrid model capable of extracting phrases representing an arbitrarily long document in the Slovak language.

## Approaches for sentiment analysis in financial domain

Sentiment analysis is a computational technique used to identify and categorize opinions or emotions expressed within a text. In this study, we wanted to employ sentiment analysis to assess the tone of bank communications by classifying extracted keywords into positive, negative, and neutral sentiment categories. Sentiment can essentially be classified using two approaches.

### Machine learning approaches

Machine learning approaches for financial sentiment analysis leverage supervised learning algorithms, or deep learning algorithms to train models on labelled datasets. These models effectively identify intricate patterns and relationships within financial texts. However, their reliance on extensive labelled data poses a significant challenge due to the high cost and time associated with data annotation.

The unique characteristics of financial language, characterized by specialized jargon, pose significant challenges for general sentiment analysis models. Consequently, the development of domain-specific models and techniques is crucial. Furthermore, the scarcity of labelled financial data hinders the effective application of deep learning methods in this domain [21,22].

Pre-trained language models, exemplified by **FinBERT** [23], have emerged as effective solutions for the challenges inherent in financial sentiment analysis. These models, specifically fine-tuned on financial datasets, demonstrate superior performance compared to traditional machine learning approaches, even when trained on relatively smaller datasets.

For the Slovak language, there is no large selection of pre-trained models for sentiment classification from financial texts. In our recent study, we took a dataset of sentiment-tagged financial news sentences and searched for an optimal model for sentiment classification from these sentences, and Support Vector Machines proved to be the most effective [24]. However, in the context of the presented study, in which our goal is to analyse sentiment from smaller units (keywords), it is not appropriate to use these models, and therefore we decided to lean towards approaches using lexicons.

### Lexicon-based approaches

Lexicon-based approaches for sentiment analysis utilize pre-existing dictionaries that categorize words according to their sentiment (e.g., positive, negative, neutral). General-purpose lexicons assign sentiment to words. These lexicons are readily implementable and offer a rapid assessment of overall sentiment within a text. However, their limitations become evident in the financial domain, where they often fail to capture the nuances of financial terminology. To address this, domain-specific lexicons have been developed. These lexicons are tailored to financial texts and encompass terms unique to the financial domain, such as “*leverage*,” “*volatile*,” and *“stable*.” These lexicons can be created manually or through automated processes using machine learning techniques. While offering improved accuracy in financial sentiment analysis, domain-specific lexicons necessitate significant effort for their creation and ongoing maintenance.

**Loughran-McDonald (LM) lexicon** [25,26] is a specialized financial sentiment dictionary widely used in sentiment analysis of financial texts. It is designed to capture the unique language and sentiment expressions found in financial documents, making it a valuable tool for financial analysis and prediction. The LM lexicon is a cornerstone in lexicon-based sentiment analysis within the financial domain [5]. It is manually annotated by experts to effectively extract sentiment from financial texts and it had been widely used in recent studies [27–30]. In addition to the LM lexicon, several other lexicons have been employed in financial sentiment analysis. For example, the Harvard IV-4 Dictionary [31], and General Inquirer [32] are general-purpose sentiment lexicons that have occasionally been adapted for financial applications. However, these lexicons lack the domain-specific vocabulary necessary to fully capture the nuances of financial sentiment, as they are not tailored to the terminology and sentiment peculiarities inherent in financial texts.

Recent studies have also explored **hybrid approaches**, such as integrating domain-specific lexicons like LM with machine learning models to enhance performance [33–35]. Although these approaches demonstrate promise, the computational overhead and potential overfitting on domain-specific data raise concerns about scalability and generalizability.

Another recent research has concentrated on the **automated construction of financial sentiment lexicons** to mitigate the scarcity of domain-specific resources. Techniques such as weighted Pointwise Mutual Information and context-aware methods have been utilized to create lexicons that effectively incorporate negation and contextual nuances, thereby enhancing the accuracy of sentiment classification within the financial domain [34].

A significant challenge in developing financial sentiment lexicons lies in the context-dependent nature of financial language. The sentiment associated with a particular word can vary significantly depending on its surrounding words. To address this, context-aware lexicons, such as **Senti-DD**, have been developed. These lexicons incorporate direction-dependent words, recognizing that the sentiment of a word can be influenced by the words that precede or follow it [36].

Despite these alternatives, the LM lexicon remains the preferred choice. Even if lexicon-based approaches, such as the LM lexicon, rely on static, predefined word lists and sentiment scores, which limit their adaptability to context, in our case we are classifying keywords consisting of unigrams and bigrams so the context would not get lost.

## Materials and methods

This chapter is dedicated to the description of the methodology. It describes the collection of data from which keywords were extracted (our corpus), the proposed extraction model and post-processing to create the final dataset.

### Data collection

We started with the collection of textual data. In the corpus, we included the texts of the commercial bank documents related to mandatory disclosure under Pillar 3, while the object of our interest were primarily those parts of the documents that contain non-tabular, unstructured or semi-structured information. This was done using Python programming language along with the Pdf plumber library [37] to automate this process. Pdf plumber is a Python library that makes it easy to work with PDF files. It allowed us to extract only textual data from PDF files. We have divided the documents into two large groups according to [2], namely Pillar 3 disclosure requirements, which are information that are mandatorily disclosed under Pillar 3. The second group are the documents disclosed under other parts of regulation, such as accounting rules or financial markets regulations, and provide valuable insight into risk profile and management of the banks. Specifically, these documents include annual reports, annual financial reports, half-yearly financial reports and covered bonds issuers related reports.

Annual reports have been available since 2007, up to the year 2022. The structure of annual reports has undergone slight changes over the years. Chapters that remained constant in terms of structure included the Chairman of the Supervisory Board’s Foreword, the Chairman of the Management Board’s Foreword, External Environment Development, Bank’s Results for the Respective Year, Evaluation of Economic and Financial Situation, and Information on the Expected Economic and Financial Situation in the Following Year.

The analysed financial reports are those that are reported by the bank in connection with the Securities Act, as the issuer of the share and they have been available since 2017. For these documents, chapters remained unchanged over the years. Each document consisted of three chapters, excluding the Accounting Closure chapter due to its structured nature. The following chapters were considered: Issuer Identification and Annual Report.

Semi-annual financial reports, issued on the basis of capital marker regulation, have been available since 2018. Similar to annual financial reports, chapters remained unchanged for these documents.

Information on the bank’s activities, which forms a part of Pillar 3 framework, includes textual information and has been available quarterly since 2020. From this information, the following chapters were of interest: Introduction, Requirements according to NBS Regulation No. 16/2014 in force, Disclosure requirements according to Part Eight of Regulation (EU) No. 575/2013, and Statement by the manager responsible for preparing the company’s financial reports.

Prospectuses belonging to covered bonds, issued on the basis of the covered bonds regulation, are the basis for the examined documents. These have been available since 2016, and the following chapters were included in our corpus: Risk Factors, Statements of Responsibility, Documents Included by Reference, Issuer Information, Use of Proceeds, and Description of the Slovak Covered Bonds Framework.

### Keyword extraction

**Fig 1:**
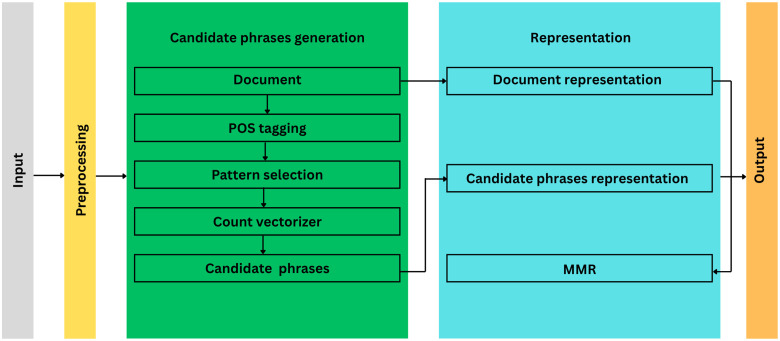
Architecture of proposed hybrid model for keyword extraction.

In the Approaches to keyword extraction section, we discussed statistical, linguistic, machine learning and hybrid approaches to keyword extraction. We found that statistical approaches are language-independent, but they are not the most suitable solution for inflectional languages. Linguistic approaches and machine learning approaches, on the other hand, are language-dependent and therefore, in the case of adaptation to a specific language, they can provide an effective solution. Our proposed hybrid model uses elements of statistic approaches (frequency counting), linguistic approaches to the selection of candidate phrases (noun phrases) and then calculates the similarity to the source document for these phrases and, like KeyBERT, implements MMR to ensure the required diversity among the selected phrases. The architecture of the used model is shown in Fig 1. The architecture consists of the input unit, preprocessing unit, module of candidate phrases generation, representation module and output unit.

The input unit receives a document along with the desired range of n-grams and the number of keywords to be extracted by the model. Additionally, it allows for specifying the diversity of keywords per position, ranging from 0 to 1. In our scenario, we grouped documents based on chapters and extracted keywords separately for each chapter. For every chapter, we extracted 50 bigrams and 50 unigrams with a diversity of 0.8, ensuring the diversity of the resulting set of keywords. Keywords we were expecting were phrases like *covid-19*, *finančná kríza (financial crisis)*, *pákový efekt (leverage effect)*, etc. However, as the degree of n-gram increases, the frequency of occurrence and thus the “keyness” itself would decreased. This is the reason why we selected unigrams and bigrams. The preprocessing unit includes sentence tokenization and lowercasing. Stop words and punctuation are not removed in this phase. This decision is made because in subsequent phases, we will select patterns of co-occurring n-grams based on post tags. Removing stop words or punctuation at this stage could result in the extraction of patterns that do not occur together.

### Candidate phrase generation

Traditional keyword extraction algorithms based solely on statistical methods, such as RAKE, are language-independent. However, when applied to Slovak using only frequently occurring n-grams, the extracted phrases may lack meaningful interpretation. Additionally, statistical approaches require extensive preprocessing, including tokenization, removal of punctuation, special characters, and stop words, to improve extraction accuracy. Machine learning-based keyword extraction methods, which often rely on measuring cosine similarity between n-grams and the document, can be computationally expensive and do not always guarantee meaningful phrase extraction. Furthermore, many machine learning models have constraints on the number of tokens they can process, making them more suitable for extracting keywords from short texts rather than longer documents. To address these challenges, our proposed model begins by tagging the entire document with Universal Part-of-Speech (UPOS) tags using the Stanza library [38]. This tagging process identifies not only parts of speech but also punctuation marks, eliminating the need for extensive preprocessing. Instead of manually filtering stop words, we automatically exclude all words that receive a UPOS tag, except for specific patterns representing noun phrases. For unigram keyword extraction, we retain only nouns. For bigrams, we extract meaningful phrase structures, specifically combinations of two consecutive nouns or an adjective followed by a noun. These extracted noun phrase patterns form the basis of our keyword vocabulary, which is then used for statistical representation in a bag-of-words model. By focusing on linguistically valid phrase structures, our approach ensures the extraction of more meaningful candidate phrases compared to purely statistical or machine learning-based methods.

### Document and phrase level representation

There is not a large number of pre-trained language models for the Slovak language, as for example for the English language. One option is to use the BERT multilingual base model [39], which is pre-trained on 104 languages, including Slovak. Unfortunately, the Slovak portion of the dataset is relatively small compared to high-resource languages like English. The exact size is not explicitly detailed in the original publication.

Another option is XLM-RoBERTa [40]. This is a multilingual model trained on 100 languages, including Slovak. It is based on the RoBERTa architecture and is known for its strong performance across various languages and tasks. The Slovak part of the XLM-RoBERTa model was trained using the CommonCrawl corpus.

A BERT based model named SlovakBERT [41] was trained specially for Slovak language. The training data consisted of 19.35 GB of text includes Slovak Wikipedia, Open Subtitles, and OSCAR datasets. The model is specifically tailored for Slovak language, which could indicate deeper understanding of the language’s grammar, syntax, and nuances compared to multilingual models like mBERT or XLM-RoBERTa. It is likely to produce more accurate and contextually relevant embeddings for Slovak text, as it is not diluted by the need to generalize across multiple languages. These are the reasons why we used SlovakBERT over the previously mentioned models.

A representation of the input document is created as an average of the sentence embeddings of the document. Both the phrase representation and the document have an embed size of 768.

**Fig 2:**
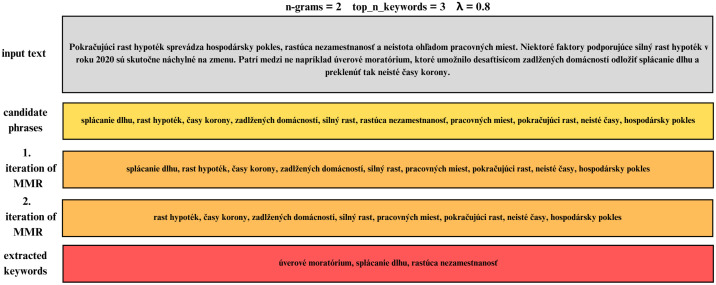
Illustration of selecting the most diverse keywords.

The maximum marginal relevance (MMR) component serves to maximize the overall informativeness of the extracted keywords. The flow of algorithm is illustrated on Fig 2. The diversification parameter on the input affects the diversity of the resulting keywords. The MMR score is calculated by combining the similarity score with a diversity term, which ensures that the selected keywords are not only relevant to the document, but also different from each other. In each iteration, the component selects the keyword with the highest MMR score, adds it to the final set of keywords, and then removes it from the pool of remaining candidates. This process continues until the specified number of keywords is reached or until there are no candidates left. Using MMR prevents very similar keywords from dominating the final selection, which contributes to a more diverse and representative set of keywords.

### Post-processing

Our goal was to look at the evolution of keywords in terms of sentiment. First of all, we needed to capture the frequency of each unique keyword within the chapter in individual years. Due to the fact that Slovak is an inflectional language, i.e., keywords can have different endings, we applied lemmatization to individual keywords and also lemmatized our corpus. Lemmatization was done using aforementioned Stanza library [38]. Subsequently, determined the frequency of the keyword in the chapter under study (the number of its occurrences). The final step was tagging keywords with sentiment. Existing pre-trained models that can be used for sentiment classification in the Slovak language are intended for determining sentiment from sentences or broader texts. Since our goal is to determine the sentiment of the extracted keywords, we have chosen a financial lexicon-based approach. In our sentiment analysis, we included neutral words alongside positive and negative words to provide a comprehensive understanding of the communication strategies employed by Slovak banks. Neutral words are essential for following reason. Neutral words are common in technical and financial reports, where the goal is to present information in a factual and objective manner. Analysing neutral words helps us understand how banks maintain transparency and objectivity in their communications, especially in documents like financial reports and covered bonds.

**Fig 3:**
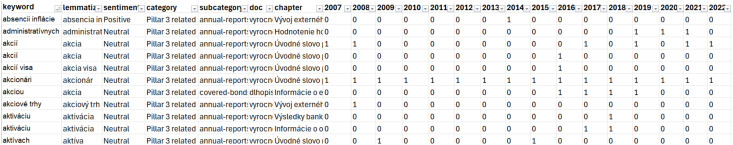
Resulting dataset after keyword extraction, sentiment classification and frequency analysis.

Given that our lemmatized keywords were in Slovak and the LM lexicon is in English, we translated the keywords into English and tagged them using the LM lexicon. The extracted keywords with assigned sentiment were then manually checked. As an example, we present the keyword *akcia (action)*, which is classified as neutral, the keyword *absencia inflácie – (absence of inflation)*, which is classified as positive, or the keyword *ekonomická kríza – economic crisis* which is classified as negative. A sample of the resulting dataset is present at Fig 3.

After creating the dataset, we saw interesting results. For example, during the financial crisis, keywords such as *loan* or *decline* were mentioned. During the third pillar review, we recorded “regulated market”, *Basel Committee*, *regulatory regulation* as key topics. During the COVID-19 pandemic, the extracted keywords included *corona*, *covid-19 pandemic*, *crisis*. During the energy crisis and the war in Ukraine, the topic focused on key phrases such as *Ukraine*, *Russian ruble*.

### Computational resources

The implementation of our hybrid model was carried out using a high-performance computing environment. Our server has AMD EPYC 7542 32-Core Processor, and 377 GB RAM. No GPU was used. These resources ensured efficient processing and analysis of the large volume of textual data.

### Statistical analysis and visualization of results

To analyse the sentiment of keywords across financial reports, various statistical tests were employed. For repeated measures, Mauchly’s test of sphericity was used to assess whether the assumption of sphericity in the covariance matrix was met. In cases where sphericity was violated, adjustments were made using Greenhouse-Geisser corrections to ensure valid comparisons across time periods. Unadjusted and adjusted univariate tests for repeated measures were applied to determine if the frequency of sentiment-labelled words varied significantly over time.

Additionally, multilevel comparisons were performed to examine the differences in sentiment distribution across distinct economic periods. These comparisons helped reveal how banks’ communication strategies evolved during financial crises, regulatory changes, and periods of stability. These statistical methods help confirm whether changes in sentiment distribution were dependent on economic events.

To illustrate the trends in sentiment expression within financial reports, graphical methods were employed. Specifically, line charts were used to depict the evolution of positive, negative, and neutral keywords across different economic periods. These visualizations allow for an intuitive understanding of how sentiment fluctuates in response to economic crises, regulatory changes, or financial instability.

## Results

We hypothesize that the frequency of positive/negative/neutral sentiment words extracted from the studied documents depends on time. Regardless of the category of the document, the most negative words were presented during the COVID-19 pandemic – 581, followed by Pillar 3 revision – 404, the period after the GFC – 351, GFC – 132, Ukraine war – 100, and before the crisis period – 32.

Neutral keywords were used very often regardless of the turbulent period, which may be a consequence of the banks’ efforts to maintain a certain degree of professionalism and conservatism in presenting their results in disclosures. The highest number of neutral keywords was during the period of the energy crisis and war in Ukraine – 1769, followed by Pillar 3 revision – 1393, COVID-19 pandemic – 7701, period after GFC – 5457, GFC – 1410, and period before GFC – 1110.

The most positive keywords were presented during the after-crisis period – 569, followed by Pillar 3 revision – 525, COVID-19 pandemic – 427, before the crisis period – 127, and energy crisis and Ukraine war – 113.

In order to find out whether the frequencies of keywords with a specific sentiment category (positive, negative, neutral) depend on the observed period, as well as the category of the document, we performed statistical tests (Tables 1–12).

**Table 1:** Mauchly’s test of sphericity for the frequency of negative words across the observed years.

	W	Chi-Sqr.	df	p
covered-bonds: YEAR	0.531	16.438	2	**0.0003**
annual-reports: YEAR	0.980	5.025	2	0.0810
financial-reports: YEAR	0.461	14.717	2	**0.0006**
information-about-bank: YEAR	0.642	12.391	2	**0.0020**

**Table 2:** Mauchly’s test of sphericity for the frequency of positive words across the observed years.

	W	Chi-Sqr.	df	p
covered-bonds: YEAR	0.565	6.281	2	**0.0433**
annual-reports: YEAR	0.838	52.986	2	**0.0000**
financial-reports: YEAR	0.103	38.673	2	**0.0000**
information-about-bank: YEAR	0.687	7.499	2	**0.0235**

**Table 3:** Mauchly’s test of sphericity for the frequency of neutral words across the observed years.

	W	Chi-Sqr.	df	p
covered-bonds: YEAR	0.122	489.566	2	**0.0000**
annual-reports: YEAR	0.854	253.341	2	**0.0000**
financial-reports: YEAR	0.349	315.548	2	**0.0000**
information-about-bank: YEAR	0.488	219.528	2	**0.0000**

**Table 4:** Unadjusted univariate tests for repeated measures – frequency of negative words across observed years.

	df	F	p	G-G Epsilon	G-G Adj. Df1	G-G Adj. Df2	G-G Adj. P
covered-bonds: YEAR	–	–	–	0.681	1.362	36.770	0.4313
annual-reports: YEAR	2	5.953	**0.0028**	–	–	–	–
financial-reports: YEAR	–	–	–	0.650	1.299	25.989	0.1550
information-about-bank: YEAR	–	–	–	0.737	1.473	42.722	0.4081

**Table 5:** Adjusted univariate tests for repeated measures – frequency of positive words across observed years.

	G-G Epsilon	G-G Adj. Df1	G-G Adj. Df2	G-G Adj. P
covered-bonds: YEAR	0.697	1.394	16.724	0.9241
annual-reports: YEAR	0.861	1.721	518.116	**0.0065**
financial-reports: YEAR	0.527	1.054	18.975	0.3964
information-about-bank: YEAR	0.762	1.524	31.996	0.2182

**Table 6:** Adjusted univariate tests for repeated measures – frequency of neutral words across observed years.

	G-G Epsilon	G-G Adj. Df1	G-G Adj. Df2	G-G Adj. P
covered-bonds: YEAR	0.533	1.065	249.243	0.2042
annual-reports: YEAR	0.873	1.745	2804.699	0.1776
financial-reports: YEAR	0.606	1.212	364.694	**0.0218**
information-about-bank: YEAR	0.661	1.323	406.089	**0.0115**

**Table 7:** Multilevel comparison for the frequency of negative words in the observed years: a) Covered bonds, b) Annual reports, c) Financial reports, d) Information about bank.

a)			
**YEAR**	**Mean**	**1**	
y2019	0.714	****	
y2021	0.857	****	
y2020	0.929	****	
**b)**			
**YEAR**	**Mean**	**1**	**2**
y2019	0.232	****	
y2021	0.289	****	
y2020	0.455		****
**c)**			
**YEAR**	**Mean**	**1**	
y2021	1.476	****	
y2019	1.762	****	
y2020	2.333	****	
**d)**			
**YEAR**	**Mean**	**1**	
y2019	4.100	****	
y2020	4.333	****	
y2021	4.600	****	

**Table 8:** Multilevel comparison for the frequency of positive words in the observed years: a) Covered bonds, b) Annual reports, c) Financial reports, d) Information about bank.

a)			
**YEAR**	**Mean**	**1**	
y2021	1.769	****	
y2019	1.846	****	
y2020	1.846	****	
**b)**			
**YEAR**	**Mean**	**1**	**2**
y2020	0.172	****	
y2021	0.179	****	
y2019	0.265		****
**c)**			
**YEAR**	**Mean**	**1**	
y2020	0.842	****	
y2021	1.526	****	
y2019	1.842	****	
**d)**			
**YEAR**	**Mean**	**1**	
y2021	4.909	****	
y2019	5.409	****	
y2020	5.500	****	

**Table 9:** Multilevel comparison for the frequency of neutral words in the observed years: a) Covered bonds, b) Annual reports, c) Financial reports, d) Information about bank.

a)			
**YEAR**	**Mean**	**1**	
y2021	1.953	****	
y2019	2.183	****	
y2020	2.226	****	
**b)**			
**YEAR**	**Mean**	**1**	
y2020	0.453	****	
y2021	0.495	****	
y2019	0.501	****	
**c)**			
**YEAR**	**Mean**	**1**	**2**
y2020	2.026	****	
y2021	2.315	****	****
y2019	2.536		****
**d)**			
**YEAR**	**Mean**	**1**	**2**
y2019	5.708		****
y2021	6.143	****	
y2020	6.260	****	

We examined the sentiment of keywords at the level of subcategories in financial reports. To verify the assumption of sphericity of the covariance matrix for repeated measures across individual subcategories and observed years concerning the frequency of words with sentiment levels, Mauchly’s test was employed.

In the case of negative sentiment (Table 1), we reject the null hypothesis of sphericity for the Covered Bonds, Financial Reports, and Information About Banks subcategories at the alpha level of 0.05. However, we do not reject the null hypothesis of sphericity for Annual Reports (*p *= 0.08). Due to the violation of the assumption of sphericity of the covariance matrix, adjusted tests for repeated measures are necessary.

Mauchly’s test results indicate that sentiment expression patterns across different financial disclosures vary significantly over time, reinforcing the need for statistical adjustments to properly interpret trends. This supports the hypothesis that banks strategically adjust their sentiment tone in response to economic fluctuations.

For positive (Table 2) and neutral (Table 3) sentiment, sphericity was violated in all subcategories, indicating that word frequency distributions were not uniform over time. This result supports the need for adjusted statistical tests to account for variability.

Subsequently, we examined the results of unadjusted and adjusted univariate tests for repeated measures. The following table (Table 4) presents the results of unadjusted and adjusted univariate tests for repeated measures, analysing the frequency of negative words in the observed years for each document subcategory. We reject the null hypothesis of independence of the frequency of negative words in a specific document subcategory from the year only in the case of the Annual reports subcategory.

The following tables describe the results of adjusted univariate tests for repeated measures, analysing the frequency of positive words (Table 5) and the frequency of neutral words (Table 6) across the observed years for each document subcategory. In the case of positive words, we reject the null hypothesis of independence of word frequency in a specific document subcategory from the year, specifically in the case of the Annual reports subcategory.

For neutral words (Table 6), we reject the null hypothesis for the subcategories Financial Reports and Information About Bank.

To further quantify the significance of sentiment differences, we performed multilevel comparisons. The results indicate statistically significant differences (*p* < 0.05) in sentiment word distributions across years (Table 7). Regarding the frequencies of words with negative sentiment, statistically significant differences were found only in the case of the subcategory Annual reports, with the highest proportion of negative words occurring in 2020, during the COVID-19 pandemic. The reason behind why significant differences were found only in annual reports could be that sentiment differences emerge as a reflection of the comprehensive narrative and qualitative information they provide, setting them apart from other financial disclosures. These reports serve as a primary communication tool with stakeholders, offering detailed discussions on performance, strategic initiatives, and future outlooks, which inherently carry the sentiment of the management and the prevailing economic environment.

In the case of positive sentiment (Table 8), statistically significant differences were found again for the subcategory Annual reports, with the highest positivity being exhibited during the period of stability, namely during the review of the third pillar.

While neutral sentiment remains the dominant category in many financial disclosures, its role in financial reporting deserves further exploration. The high frequency of neutral words during crises may reflect banks’ efforts to present stability and reliability to stakeholders, mitigating panic during uncertain periods. A closer qualitative inspection of neutral sentiment reveals that these words are often used to frame discussions around “risk management,” “financial stability,” and “long-term strategy,” suggesting that banks deliberately employ neutral language to reassure investors and maintain credibility in crisis periods. In the case of neutral words (Table 9), a statistically significant difference was observed in the category Financial reports between the years 2019 and 2020, as well as in the case of the subcategory Information about bank in the years 2019 and 2020–2021. For both types of reports, the highest proportion of neutral words was observed in 2020.

**Fig 4:**
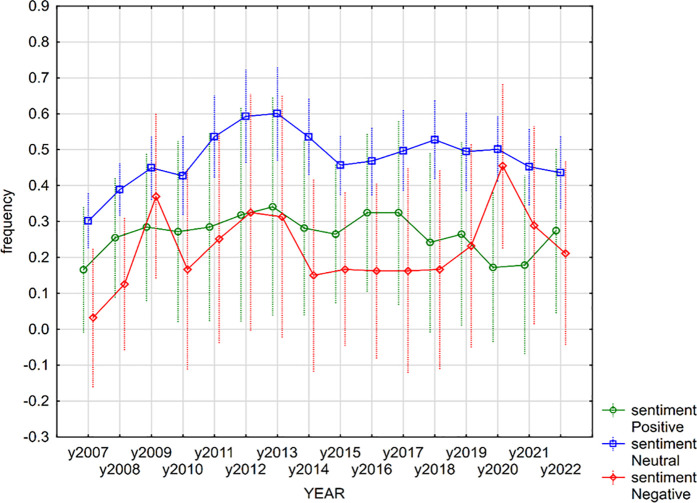
The evolution of sentiment in annual reports.

Since statistically significant differences were observed only in the case of annual reports for both positive and negative keywords, we focused our subsequent analyses specifically on annual reports. Fig 4 visualizes the evolution of the frequency of positive, negative, and neutral keywords in annual reports. From the graph, it can be observed that positive and neutral keywords had the highest frequency during periods of stability, while negative keywords had the highest frequency during periods of crisis.

The following table (Table 10) presents the results of the Mauchley test of sphericity for the word frequency in the monitored years in Annual reports in terms of sentiment. Specifically, it analyses whether the assumption of sphericity is met for different types of sentiment (negative, positive, neutral) over the monitored years. The assumption of sphericity is not met for any of the types of sentiment (negative, positive, neutral) in the monitored years. This means that statistically significant differences (*p* < 0.05) are observed in the variability of word frequencies in these sentiment categories.

**Table 10:** Mauchley Sphericity Test for the frequency of words in the examined years in terms of sentiment.

	W	Chi-Sqr.	df	p
Negative: YEAR	0.0001	2142.071	119	**0.0000**
Positive: YEAR	0.0163	1216.864	119	**0.0000**
Neutral: YEAR	0.0023	9715.607	119	**0.0000**

These statistical findings indicate that banks do not use sentiment terms uniformly but instead adapt their messaging strategies based on financial and regulatory circumstances. This insight aligns with previous studies on financial sentiment analysis in other economies, such as the U.S. Fortune 1000 companies [6] where firms approaching financial distress altered their sentiment tone. Similarly, research on Turkish, Slovak, and Ukrainian companies [8] found that negative sentiment spikes correspond to economic downturns, supporting the global relevance of our findings.

The following table (Table 11) describes adjusted univariate tests for repeated measures describing the frequency of the examined words in the monitored years in terms of sentiment. Based on the results, we can reject the global null hypothesis of independence of the sentiment category of keywords from time for all sentiment categories.

**Table 11:** Adjusted Univariate Tests for Repeated Measure – the frequency of the examined words in the observed years in terms of sentiment.

	G-G Epsilon	G-G Adj. Df1	G-G Adj. Df2	G-G Adj. P
Negative: YEAR	0.329	4.937	1209.478	**0.0000**
Positive: YEAR	0.625	9.378	2822.724	**0.0005**
Neutral: YEAR	0.481	7.209	11584.550	**0.0000**

Table 12 displays a multi-level comparison of the frequency of examined words in the monitored years based on sentiment categories: negative, positive, and neutral. For each sentiment category, the table presents the mean frequency of the examined keywords across the years within each group.

**Table 12:** Multilevel analysis for the frequency of the investigated words in the monitored years in terms of sentiment: a) Negative, b) Positive, c) Neutral.

a)							
**YEAR**	**Mean**	**1**	**2**	**3**	**4**	**5**	**6**
y2007	0.033	****					
y2008	0.126	****	****				
y2014	0.150		****				
y2017	0.163		****	****			
y2016	0.163		****	****			
y2015	0.167		****	****			
y2010	0.167		****	****			
y2018	0.167		****	****			
y2022	0.211		****	****	****		
y2019	0.232		****	****	****		
y2011	0.252		****	****	****	****	
y2021	0.289			****	****	****	
y2013	0.313				****	****	
y2012	0.325				****	****	
y2009	0.370					****	****
y2020	0.455						****
**b)**							
**YEAR**	**Mean**	**1**	**2**				
y2007	0.166	****					
y2020	0.172	****					
y2021	0.179	****					
y2018	0.242	****	****				
y2008	0.255	****	****				
y2015	0.265	****	****				
y2019	0.265	****	****				
y2010	0.272	****	****				
y2022	0.275	****	****				
y2014	0.281	****	****				
y2011	0.285	****	****				
y2009	0.285	****	****				
y2012	0.318		****				
y2016	0.325		****				
y2017	0.325		****				
y2013	0.341		****				
**c)**							
**YEAR**	**Mean**	**1**	**2**	**3**	**4**	**5**	**6**
y2007	0.302	****					
y2008	0.389		****				
y2010	0.427		****	****			
y2022	0.436		****	****			
y2009	0.450		****	****	****		
y2021	0.453		****	****	****		
y2015	0.457		****	****	****		
y2016	0.468			****	****	****	
y2019	0.495			****	****	****	
y2017	0.497			****	****	****	
y2020	0.501			****	****	****	
y2018	0.527				****	****	****
y2014	0.535					****	****
y2011	0.536					****	****
y2012	0.593						****
y2013	0.601						****

Our findings support the hypothesis that the frequency of words with different types of sentiment depends on the monitored years and document subcategories. These results have potential implications for understanding the dynamics of sentiment in text and may be useful for analyzing annual reports in historical contexts.

Observations from 2022, particularly regarding the Ukraine war and the energy crisis, align with previous trends. However, compared to the COVID-19 pandemic, where negative sentiment was predominant, disclosures in 2022 exhibited a more balanced mix of neutral and negative sentiment, possibly reflecting differing levels of uncertainty and risk perception.

For bank management, it follows that since annual reports are an important communication tool, the wording of which can have a significant impact on stakeholder behavior towards the bank, their styling is very important.

## Discussion

Our study findings contribute to the growing body of literature on sentiment analysis in financial reporting, particularly for languages with complex morphological structures, such as Slovak.

Our research aligns with previous sentiment analysis studies in financial disclosures, such as Kubaščíková et al. [8], who examined Slovak, Turkish, and Ukrainian corporate reports. Their study confirmed that positive sentiment in Slovak reports correlated with economic growth, supporting our observation that banks communicate more positively in stable periods. Additionally, our findings resonate with Dong et al. [5], who analyzed annual reports from banks globally, demonstrating that regulatory standardization impacts linguistic patterns. While their study did not focus on sentiment shifts during crises, our analysis expands on their work by showing how Slovak banks adapted their sentiment in response to financial instability, particularly during the global financial crisis (GFC) and COVID-19 pandemic.

Previous research, such as the study by Kubaščíková et al. [8], employed lexicon-based sentiment classification using LIWC2015. Our approach, while also lexicon-based, differs by implementing a hybrid keyword extraction model that prioritizes contextually meaningful terms. This allows for more precise sentiment analysis compared to traditional lexicon-based models that do not account for term importance. Unlike prior studies that focused primarily on English-language reports, our research demonstrates the applicability of sentiment analysis in the Slovak banking sector, contributing to underexplored financial domains.

While our hybrid extraction model improves keyword selection by integrating statistical, linguistic, and machine-learning techniques, it has inherent limitations. One notable constraint is the reliance on predefined part-of-speech (POS) tagging patterns. Thanks to this tagging, we extract meaningful noun-phrases, but in the future we would like to improve the model so that it puts the extracted keywords into a basic form. In case of scalability to the other languages, it is possible but few adjustments in code are needed. First of all, it is necessary to change the first parameter in the source code in the stanza model, which sets the language in the Pipeline. In our case, it is set to *sk*. In case we would like to apply the model to English data, it is necessary to change it to *en*. The second modification that needs to be made in the source code is to replace the text representation model (in our case SlovakBERT) with another model that can represent the desired language. Speaking about the limitations of our study, in the case of sentiment classification, we find a limitation in the use of lexicon-based approaches. Although this is not a limitation in the financial domain, for other domains there is a risk that we would not find a sufficiently comprehensive lexicon.

## Conclusion

In the experimental design, our focus was on analysing sentiment through the frequency of key words in different document subcategories and monitored years. The documents were selected on the basis of the level of stakeholder interest in their content (covered bonds, annual reports – without financial reports, financial reports, information about bank). The type of these documents also determines their content structure as well as the way they are worded. While annual reports are a summary document focusing on key activities and the resulting results achieved over the annual reporting period, expressed quantitatively and interpreted qualitatively, financial reports, information about bank, and covered bonds are more technocratic in nature in terms of commenting on their quantitative results.

The above document characteristics are also reflected in our analyses of communication strategies in different periods. While annual reports identify either positive shares or negative shares of word frequency in certain periods, neutral shares are typical for financial reports, covered bonds, and information about banks. The analyses also confirmed that statistically significant differences in annual reports in terms of positive word frequency are between the crisis periods representing 2020 and 2021 and the stability years 2012, 2013, 2016 and 2017, with the largest proportion of negative words in the annual reports in 2020. This increase in negative sentiment likely reflects the heightened uncertainty and challenges faced by banks during these times.

This supports our hypothesis that the frequency of positive, negative, and neutral sentiment in bank communications varies depending on the document type and the year of publication, particularly during periods of economic turbulence.

This shows that individual stakeholders can extract different information from the text of the annual reports and draw their own conclusions based on deduction in relation to the bank.

Additionally, we found that in the case of positive sentiment, the frequency of positive key words was highest during periods of stability, in particular for the years 2013, 2016 and 2017, while it decreased during the pandemic. The same trend was observed for neutral words.

Annual reports showed to be the most relevant source on which our hypothesis was statistically significant. This is understandable and could be expected as the annual reports include the most textual information and dedicated sections aiming to provide qualitative information, compared to other used reports which are more oriented to the provision of numerical information. Our study also reflects on the effectiveness of annual reports in communicating stability or risk, particularly during varying economic conditions. We observe that during periods of economic stability, annual reports tend to emphasize positive achievements and growth, which can effectively convey a sense of stability and confidence to stakeholders. However, during turbulent times, such as the Global Financial Crisis or the COVID-19 pandemic, the increased use of negative or neutral sentiment may indicate caution and uncertainty. This shift in sentiment can serve as a signal of potential risks, allowing stakeholders to assess the bank’s resilience and preparedness in challenging times. By analyzing these sentiment trends, we aim to provide insights into how banks can enhance their communication strategies to better balance the portrayal of stability and risk, ensuring transparency and fostering trust among stakeholders.

Overall, our findings support the hypothesis that the frequency of words with different types of sentiment depends on the monitored years and document subcategories. These results have potential implications for understanding the dynamics of sentiment in text and may be useful for analysing annual reports in historical contexts.

For bank management, it follows that since annual reports are an important communication tool, the wording of which can have a significant impact on stakeholder behaviour towards the bank, their styling is very important.

Our research contributes on a theoretical level by analysing related studies dealing with sentiment analysis in finance and points out their shortcomings and limitations. On a practical level, the results of the statistical analysis are the basis for the selected commercial bank. The fact that there is an association between sentiment and the gap may indicate that sentiment could be used as a predictor for modelling financial distress. The results of the analysis can also be used by bank managers to improve their future communication strategy. To illustrate why it is important we provide following examples. For instance, investors may view a predominance of positive sentiment during stable economic periods as an indicator of growth and confidence, potentially influencing their investment decisions. Conversely, a shift towards negative sentiment during crises could signal caution, prompting stakeholders to seek further clarification or reassurance from the bank. Regulators might use sentiment analysis to monitor changes in risk perceptions and market confidence, informing their oversight and policy responses. Additionally, bank management can leverage these insights to refine their communication strategies, ensuring that their messaging aligns with stakeholder expectations and effectively conveys the bank’s position and outlook.

Sentiment analysis can help banks understand how their communications are perceived by stakeholders, allowing them to tailor their messaging more effectively. By identifying patterns in sentiment during different economic periods, banks can adapt their communication to better reflect stakeholder expectations and concerns. For example, during periods of economic uncertainty, banks might use more reassuring and neutral language to convey stability, while during stable periods, they might emphasize positive achievements and growth.

## Future direction

Sentiment analysis has proven to be an effective tool for bank management and regulators alike. In our future research, we would like to focus on sentiment analysis with a focus on the Visegrad Group countries and examine how banks in individual countries communicate their key themes during times of turbulence.
